# Identifying communities from multiplex biological networks by randomized optimization of modularity

**DOI:** 10.12688/f1000research.15486.2

**Published:** 2018-11-22

**Authors:** Gilles Didier, Alberto Valdeolivas, Anaïs Baudot

**Affiliations:** 1 Aix Marseille Univ, CNRS, Centrale Marseille, I2M, Marseille, France; 2 ProGeLife, Marseille, France; 3 Aix Marseille Univ, Inserm, MMG, Marseille, France

**Keywords:** Biological Networks, Multiplex, Multi-layer, Community identification, Clustering, DREAM challenge

## Abstract

The identification of communities, or modules, is a common operation in the analysis of large biological networks. The *Disease Module Identification DREAM challenge* established a framework to evaluate clustering approaches in a biomedical context, by testing the association of communities with GWAS-derived common trait and disease genes. We implemented here several extensions of the MolTi software that detects communities by optimizing multiplex (and monoplex) network modularity. In particular, MolTi now runs a randomized version of the Louvain algorithm, can consider edge and layer weights, and performs recursive clustering.

On simulated networks, the randomization procedure clearly improves the detection of communities. On the *DREAM challenge* benchmark, the results strongly depend on the selected GWAS dataset and enrichment ***p***-value threshold. However, the randomization procedure, as well as the consideration of weighted edges and layers generally increases the number of trait and disease community detected.

The new version of MolTi and the scripts used for the DMI DREAM challenge are available at: https://github.com/gilles-didier/MolTi-DREAM.

## Introduction

Biological macromolecules do not act isolated in cells, but interact with each other to perform their functions, in signaling or metabolic pathways, molecular complexes, or, more generally, biological processes. Thanks to the development of experimental techniques and to the extraction of knowledge accumulated in the literature, biological networks are nowadays assembled on a large scale. A common feature of biological networks is their modularity, i.e., their organization around communities - or functional modules - of tightly connected genes/proteins implicated in the same biological processes ^1, 2^.

*The Disease Module Identification (DMI) DREAM challenge* aims at developing a benchmark to investigate different algorithms dedicated to the identification of communities from biological networks ^3^. The challenge has been divided into two sub-challenges, to identify communities either i) from six biological networks independently, or ii) from all these networks jointly. The second sub-challenge, in particular, intend to test if some approaches can leverage complementary information from multiple networks jointly to define integrated communities. The clustering approaches proposed by the participants are assessed regarding their capacity to reveal *disease communities*, defined as communities significantly associated with genes implicated in diseases in GWAS studies ^3, 4^.

The challengers proposed various strategies and clustering approaches, including kernel clustering, random walks or modularity optimization. We competed with an enhanced version of MolTi, a modularity-based software that we recently developed ^5^. We focused on the subchallenge dedicated to the identification of communities from multiple networks as MolTi was initially developed to cluster multiplex networks, i.e., networks composed of different layers of interactions. Molti extended the modularity measure to multiplex networks and adapted the Louvain algorithm to optimize this multiplex-modularity. We have demonstrated that this multiplex approach better identifies the communities than approaches merging the networks, or performing consensus clusterings, both on simulated and real biological datasets ^5^.

Grounded on these initial results, we here extended and tested our MolTi software, both on simulated data and on the DMI challenge framework. We improved MolTi with the implementation of a randomization procedure, the consideration of edge and layer weights, and a recursive clustering of the classes larger than a given size.

With simulated data, we observed that considering more than one network layer improves the detection of communities, as already noted in Didier *et al.*, 2015 ^5^, but also that communities are better detected with the randomization procedure. With the DMI benchmark, we pointed to a great dependence on the GWAS dataset used for the evaluation and on the FDR threshold defined, but, overall, randomizations and edge and layer weights increase the number of disease communities detected.

## Methods

### MolTi-DREAM: communities from multiplex networks

We detected communities with an extended version of MolTi ^5^, a modularity-based software. Although MolTi was specifically designed for multiplex networks, (i.e., networks composed of different layers of interactions), it deals with monoplex networks (i.e. single-layer network) by considering them as multiplex networks composed of a single layer. All the networks are here considered undirected. The new version of MolTi, MolTi-DREAM, and the scripts used for the DMI DREAM challenge are available at https://github.com/gilles-didier/MolTi-DREAM.

***Modularity.*** Network modularity was initially designed to measure the quality of a partition into communities ^6^, and subsequently used to find such communities. Since finding the partition optimizing the modularity is NP-complete, we applied the meta-heuristic Louvain algorithm ^7^. This algorithm starts from the community structure that separates all vertices. Next, it tries to move each vertex from its community to another, picks the move that increases modularity the most, and iterates until no change increases the modularity any more. It then replaces the vertices by the detected communities and performs the same operations on the newly obtained graph, until the modularity cannot be increased any more. In order to handle multiplex networks, we use a multiplex-adapted modularity and an adaptation of the Louvain algorithm for optimizing this multiplex-modularity.

**Edge and layer weights** Modularity approaches can deal with weighted networks ^8^, and we modified MolTi to handle weighted networks. We also added the possibility to weight each layer of the multiplex network: the contribution of each layer in Equation (1) is multiplied by its weight when computing the multiplex modularity.

**Multiplex modularity** The modularity measure to detect communities in a multiplex network ( *X* ^( *g*)^) _*g*_ can be written as

$$\sum_{g}\frac{w^{\left(g\right)}}{2m^{\left(g\right)}}\sum_{\begin{array}{c} \left\{i,j\right\}\\ i\neq j \end{array}}\left(X_{i,j}^{\left(g\right)}-\gamma \frac{S_{i}^{\left(g\right)}S_{j}^{\left(g\right)}}{2m^{\left(g\right)}}\right)\delta_{{\text{c}}_{i},{\text{c}}_{j}},\;(1)$$

where *X* ^( *g*)^ denotes the (monoplex) network of the layer *g*, *w* ^( *g*)^ is the user-defined weight associated to the network g, *m* ^( *g*)^ is the sum of the weights of all the edges of *X* ^( *g*)^, $X_{i,j}^{\left(g\right)}$ is the weight of the edge { *i*, *j*} in *X* ^( *g*)^, $S_{i}^{\left(g\right)}$ is the sum of the weights of all the edges involving vertex *i* in *X* ^( *g*)^, *δ*_**c _*i*_**, **c _*j*_**_ is equal to 1 if *i* and *j* belong to a same community and to 0 otherwise, and γ is the resolution parameter modulating the size of the communities detected.

***Randomization.*** We implemented a randomized version of the Louvain algorithm, similar to the one in GenLouvain ^9^. Rather than updating the current partition by picking the move leading to the greatest increase of the modularity, we randomly pick a move among those leading to an increase of the modularity. Different runs of the randomized Louvain generally return different partitions, even if the results are often close. MolTi-DREAM runs the randomized Louvain algorithm a user-defined number of times, and returns the partition with the highest modularity.

### Simulations of Multiplex Networks with a known community structure

We simulated random multiplex networks with a fixed known community structure and various topological properties by using Stochastic Block Models (SBMs) as in Didier *et al.*, 2015 ^5^. SBMs model networks with a given community structure under the key assumption that all edges are drawn independently conditionally on the communities to which their nodes belong. In our simulations, we considered multiplex networks with 1,000 vertices split into 20 balanced communities. Each individual network of these multiplex networks is then simulated by independently drawing edges with fixed intra and inter community edge probabilities: 0.1 and 0.01 for sparse networks and 0.5 and 0.2 for dense ones. Dense (resp. sparse) multiplex networks contain only dense (resp. sparse) networks, while mixed networks contain both sparse and dense networks. Multiplex networks with missing data are obtained by randomly removing half of the vertices (and the edges involving them) of the multiplex networks simulated from SBMs.

The relevance of a community structure is assessed by computing the adjusted Rand index ^10^ between the detected communities and the ones used to simulate the multiplex networks.

### The Disease Module Identification challenge benchmark

***Biological Networks.*** The DMI challenge provided six human biological networks: two protein-protein interactions, one literature-curated signaling, one co-expression, one network linking genes essential for the same cancer types, and one network connecting evolutionary-related genes. These six networks have various sizes and edge densities ( Table 1). All networks have weighted edges, and all networks but the signaling network are undirected. However, we considered the signaling network as undirected.

**Table 1.** Number of vertices, of (non-zero-weighted) edges and density of the biological networks used in the DMI challenge.

Network	Number of nodes	Number of edges	Density
1-ppi	17,397	2,232,405	1.48 × 10 ^−2^
2-ppi	12,420	397,309	5.15 × 10 ^−3^
3-signal	5,254	21,826	1.34 × 10 ^−3^
4-coexpr	12,588	1,000,000	1.26 × 10 ^−2^
5-cancer	14,679	1,000,000	9.28 × 10 ^−2^
6-homology	10,405	4,223,606	7.80 × 10 ^−2^

***Evaluations with GWAS data.*** The communities identified by the different challengers were evaluated according to the associations of their member genes with GWAS data, using the PASCAL tool described in Lamparter *et al.*, 2016 ^4^. The procedure leverages the SNP-based *p*-value statistics obtained from 180 GWAS datasets, covering common diseases and traits. The communities are associated with p-values, then corrected for multiple testing, and an FDR threshold is used to determine the number of significant disease communities in a given partition ^3, 4^. We used three datasets: the “Leaderboard” (76 GWASs) and “Final” (104 GWASs), which were used during the challenge, and their union in a “Total” dataset (180 GWASs).

***Obtaining modules in a given size range.*** The DMI challenge set up two constraints on the submitted communities: no overlap and a size ranging from 3 to 100 nodes. We here post-filtered all partitions to keep only classes containing from 7 to 100 nodes.

**Resolution parameter** Modularity-based clustering approaches are often associated to a resolution parameter *γ* to tune the size of the obtained communities. We tested different values of this parameters ( *γ* = 1, *γ* = 5, *γ* = 10, *γ* = 100), but the leaderboard tests showed clearly better results for the recursive approach. We chose to keep the default *γ* = 1 and focused on this recursive procedure.

**Recursion procedure** We re-clustered all the communities above a certain size (here 100 vertices) by extracting the corresponding subgraphs from the networks and applying recursively the MolTi algorithm. We iterated the process until obtaining only communities with less than 100 vertices, if possible (some communities with more than 100 vertices cannot be split by considering modularity).

## Results

### Randomization improves community detection on simulated multiplex networks

**Figure 1.**
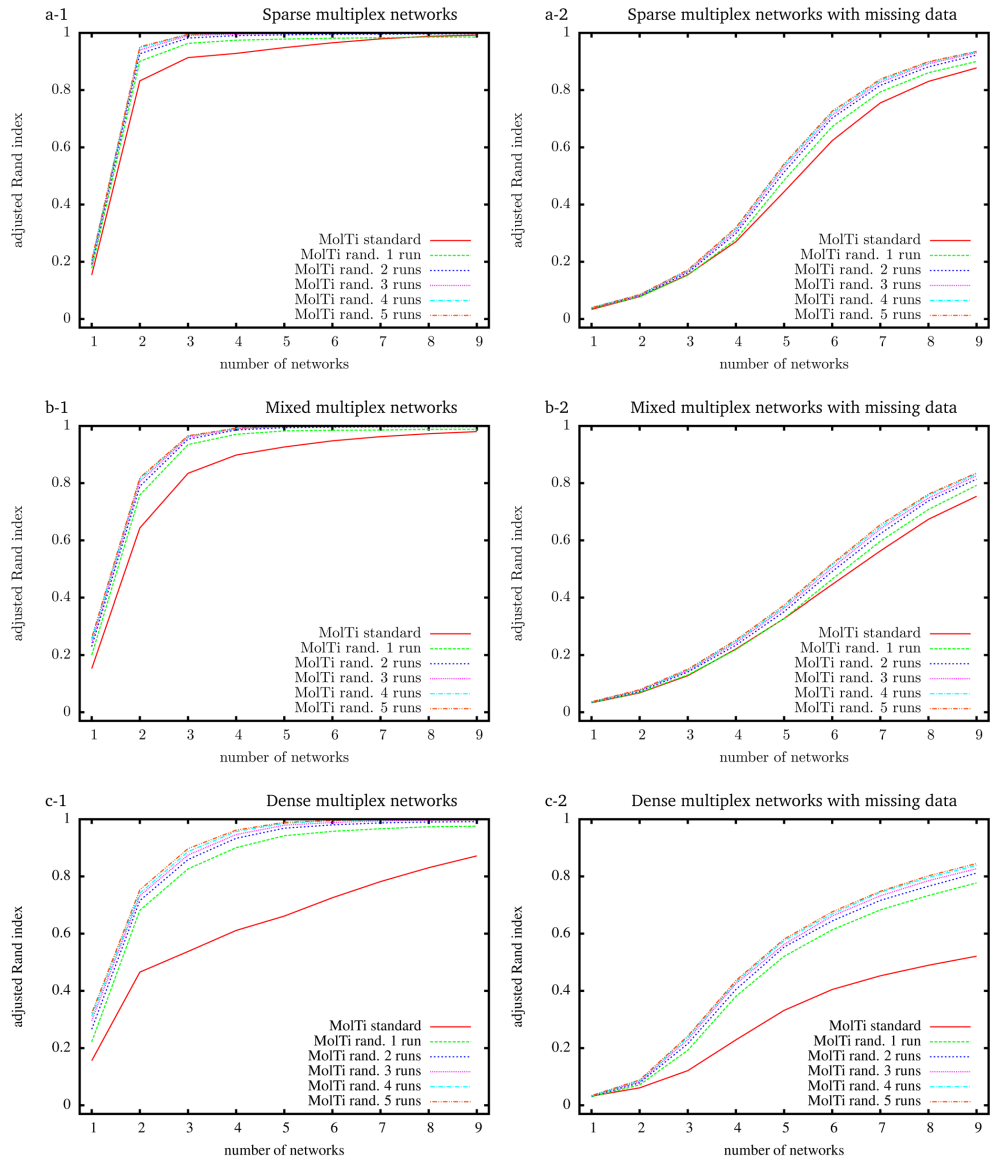
Adjusted Rand indexes between the reference community structure used to generate the random multiplex networks, and the communities detected by standard and randomized MolTi with 1 to 5 randomization runs. Multiplex networks contain from 1 to 9 graph layers. The indexes are averaged over 2,000 random multiplex networks of 1,000 vertices and 20 balanced communities. Each layer of sparse (resp. dense) multiplex networks is simulated with 0.1/0.01 (resp. 0.5/0.2) internal/external edge probabilities. Mixed multiplex networks are simulated by uniformly sampling each layer among these two pairs of edge probabilities. Multiplex networks with missing data (right column) are generated by removing vertices from each layer with probability 0.5.

To evaluate the accuracy of the community structures detected from the initial MolTi and its improved version that includes the randomization procedure, we simulated random multiplex networks with a fixed, known community structure, and various features (Methods). We observed that considering a greater number of layers always improves the inference of communities, as already observed ^5^ ( Figure 1). In addition, communities are better detected from sparse multiplex networks than from dense ones. We also observed that the randomizations improve the accuracy of the detected communities, in particular for dense multiplex networks, with or without missing data. Increasing the number of randomizations improves the results up to four randomization runs.

### Finding disease modules with MolTi

**Figure 2.**
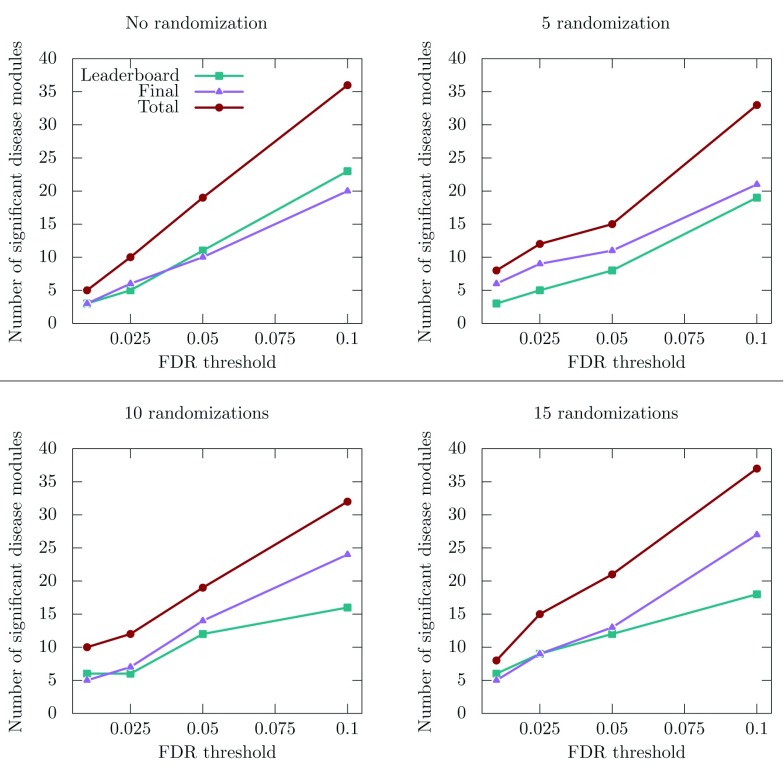
Number of significant disease modules identified from the multiplex network for different GWAS datasets and FDR thresholds. “Leaderboard” and “Final” datasets were used during the training and final evaluation of the challenge, respectively, whereas the “Total” dataset is the union of the two previous ones. The total number of considered communities is 605 in the absence of randomization, 584 for 5 randomizations, 585 for 10 randomizations and 582 for 15 randomizations.

We applied the improved MolTi to the networks provided by the DMI challenge (Methods). We focused on the sub-challenge 2, which was dedicated to the identification of communities from multiple networks. We considered the six DMI biological networks as layers of a multiplex network, and applied the recursion procedure to obtain communities in the required size range. The significant disease communities were selected regarding their enrichments in GWAS-associated genes (Methods). We observed first that the number of detected disease communities is strongly dependent on the GWAS dataset and FDR threshold used ( Figure 2). For the FDR threshold used during the challenge, i.e., FDR lower than 0.05, the number of significant disease modules detected slightly increases after randomization ( Figure 2). 

**Figure 3.**
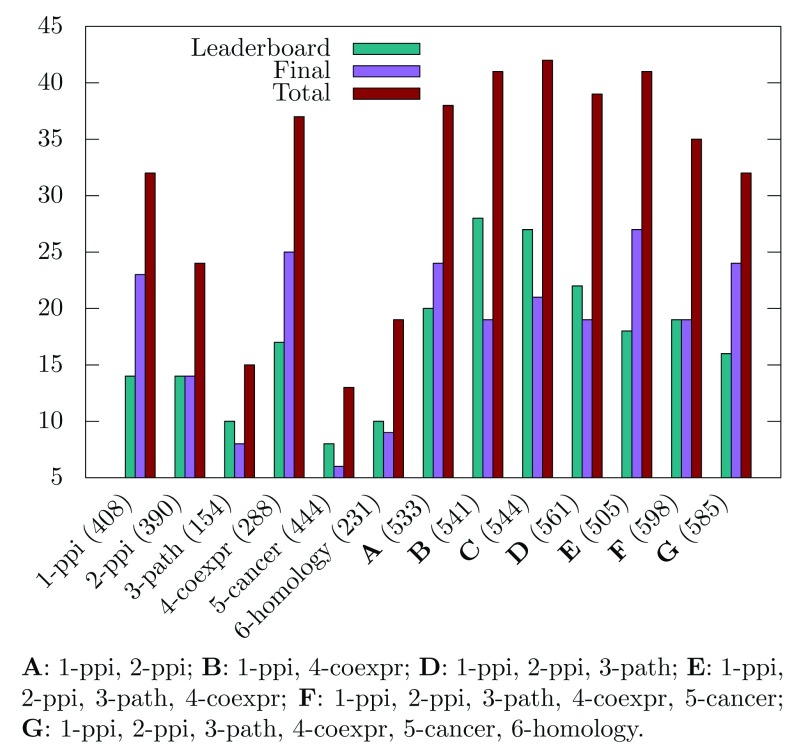
Number of significant disease modules identified for different combinations of multiplex network layers. Ten randomizations have been applied, and the FDR threshold is set to 0.1. The total number of considered communities for each multiplex network is displayed in parenthesis.

***Multiplex versus monoplex.*** We next evaluated the added value of the multiplex approach as compared to the identification of modules from the individual networks. When analyzing the significant disease modules obtained for an FDR threshold of 0.1, we observed that combining biological networks in a multiplex generally increases the number of significant modules ( Figure 3). However, this does not stand for the cancer and/or homology networks, which lower the number of significant modules retrieved when added as layers of the multiplex. We hypothesize that the community structures of these networks (if they exist) are so unrelated that it is pointless to seek for a common structure by integrating them.

These observations are consistent with the DMI challenge observations, in which the top-scoring team in the sub-challenge 2 handled only the two protein-protein interaction networks. Our algorithm also performs well with the two protein-protein interaction networks, but the highest number of disease modules is retrieved by considering network combinations that exclude the cancer and homology network layers ( Figure 3).

***Evaluation of the edge and layer weighting.*** All the six biological networks used in the DMI challenge have weighted edges. We compared the number of disease modules obtained by considering or not considering these weights in the MolTi partitioning, for different FDR thresholds ( Table 2). We observed that intra-layer edge weights only has a slight effect on the number of significant disease modules identified, except for the very low significance threshold of 0.01, where it seems pertinent to use these weights.

**Table 2.** Number of significant disease modules detected over 615 and 585 considered modules in the unweighted and weighted contexts, respectively.

FDR	Unweighted	Weighted
0.01	5	10
0.025	13	12
0.05	20	19
0.1	30	32

**Figure 4.**
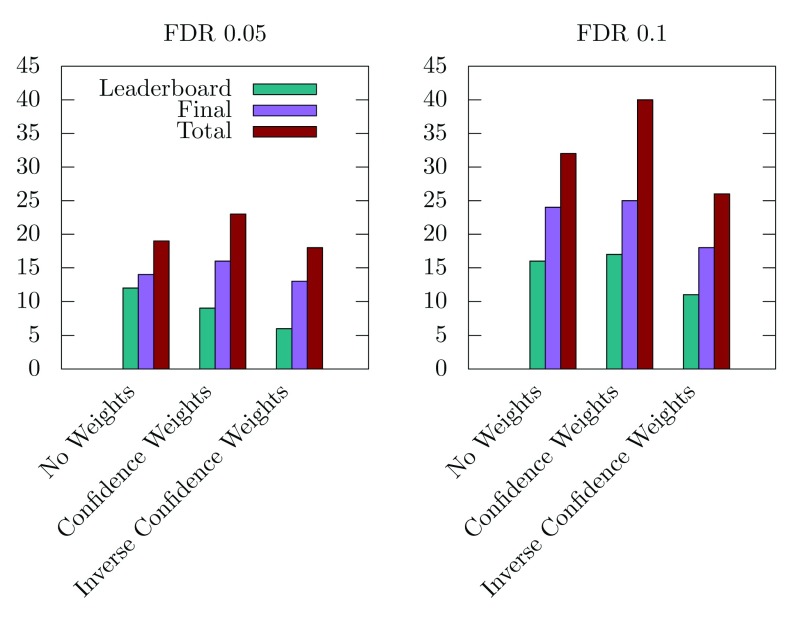
Number of significant disease modules identified with FDR thresholds 0.05 and 0.1, and from three different inter-layer weightings: *No Weights*, i.e., equal weights for all layers (585 modules in total), *Confidence Weights*, i.e., weights proportional to the expected biological relevance: 1-ppi=1, 2-ppi=1, 3-path=1, 4-coexpr=0.5, 5-cancer=0.1, 6-homology=0.1 (555 modules in total), and *Inverse Confidence Weights*, i.e., weights inversely proportional to the expected biological relevance: 1-ppi=0.1, 2-ppi=0.1, 3-path=0.1, 4-coexpr=0.5, 5-cancer=1, 6-homology=1 (648 modules in total).

MolTi-DREAM allows assigning weights to each layer of the multiplex network, for instance to emphasize the layers known to contain more relevant biological information. Given the results obtained on individual networks, we decided to test a combination of weights that would lower the importance of the 5-cancer and 6-homology network layers. We observed that this led to detecting more disease modules ( Figure 4). Conversely, less disease modules are detected when higher weights are given to these networks ( Figure 4).

## Discussion and conclusion

We applied here the MolTi software and various extensions to identify disease-associated communities following the DMI challenge benchmark. The new version of MolTi, MolTi-DREAM, runs a randomization procedure, takes into account edge and layer weights, and performs a recursive clustering of the classes that are larger than a given size. We finished tied for second in the challenge. However, even if we obtained higher scores than monoplex approaches, the difference was not significant and the organizers of the DREAM challenge declared the sub-challenge 2 vacant.

In the simulations, all the networks are randomly generated from the same community structure. These networks can thereby be seen as different and partial views of the same underlying community structure. Combining their information in a suitable way is thereby expected to recover the original structure more accurately. In contrast, combining networks with unrelated community structures (or no structure at all) is rather likely to blur the signal carried by each network. The DMI biological networks are constructed from different biological sources that might correspond to unrelated community structures. This may explain the results of the sub-challenge 2, in which the top-performer used only the two protein-protein interaction networks. With MolTi, we tried to leverage information from the 6 networks together. However, we do not obtain the highest number of disease modules from a multiplex containing all the six networks, but rather from a subset of these networks.

From a biological perspective, the protein-protein networks and the pathway networks are expected to contain mainly physical or signaling interactions between proteins. It has been shown that interacting proteins tend to be co-expressed ^11^, which could explain why the co-expression network also provides complementary information. In contrast, the cancer network is determined from processes operating at a very different level. Overall, these results show that the sources of biological information that are added as layers of a multiplex need to be evaluated thoroughly.

Evaluating the relevance of the community structure detected from real-life datasets is a very complicated problem since the actual structure is hidden and generally unknown. In this context, the only possibility for assessing the detected communities is to consider indirect evidence provided by some independent biological information. Different teams are thereby developing proxies to evaluate the communities, mainly based on testing the enrichment of genes contained in each community in Pathways or Gene Ontology annotations. The approach followed by the DMI DREAM challenge is based on GWAS data. This GWAS-based evaluation is specific in the sense that it considers *p*-value-weighted annotations rather than usual binary ones, i.e., “annotated/not annotated”. This probably contributed to the volatility of the results observed with the DMI DREAM challenge framework.

## Data availability

MolTi-DREAM and the scripts used for the DMI DREAM challenge: https://github.com/gilles-didier/MolTi-DREAM

Archived scripts and source code for MolTi-DREAM as at time of publication: http://doi.org/10.5281/zenodo.1468950 ^12^

License for MolTi-DREAM: GNU 3

## Author information

GD designed MolTi and its extensions, AB and AV applied MolTi during and after the challenge. All authors participated in the design of the study, the interpretation of the results and the writing of the manuscript.
