# Supplementary material for: Identifying communities from multiplex biological networks by randomized optimization of modularity
Source: F1000Res. 2018 Nov 22;7:1042. Originally published 2018 Jul 10. [Version 2] doi: 10.12688/f1000research.15486.2 (PMC6107982; doi:10.12688/f1000research.15486.2)
Supplement: Peer review history

# Peer review: Referee response for version 2

**DOI:** 10.5256/f1000research.18430.r41026

**Published:** 2018-12-03

**Authors:** Arda Halu

**Affiliations:** 1 Channing Division of Network Medicine, Department of Medicine, Brigham and Women's Hospital (BWH), Harvard Medical School, Boston, MA, USA

The authors have diligently addressed my comments and provided detailed discussions on the points I raised. The limitations due to being a companion paper to a flagship study considered in another journal are stated clearly and are reasonable.

I have read this submission. I believe that I have an appropriate level of expertise to confirm that it is of an acceptable scientific standard.

# Peer review: Referee response for version 2

**DOI:** 10.5256/f1000research.18430.r41029

**Published:** 2018-11-29

**Authors:** Emre Guney

**Affiliations:** 1 Research Programme on Biomedical Informatics, the Hospital del Mar Medical Research Institute, Pompeu Fabra University, Barcelona, Spain

I thank the authors for their rigorous amendments to the article and the detailed correspondence to the concerns raised by the reviewers. I only have the following minor comments:

- Gamma=1 could be added next to the Recursion label for clarity in Figures 1-3 in the response
- "We finished tied for second in the challenge." => We tied for the second place
- "," is used instead of "." to represent decimal in Tables 2-5 in the response

I have read this submission. I believe that I have an appropriate level of expertise to confirm that it is of an acceptable scientific standard.

# Peer review: Referee response for version 1

**DOI:** 10.5256/f1000research.16880.r36849

**Published:** 2018-08-17

**Authors:** Arda Halu

**Affiliations:** 1 Channing Division of Network Medicine, Department of Medicine, Brigham and Women's Hospital (BWH), Harvard Medical School, Boston, MA, USA

In their manuscript entitled “ **Identifying communities from multiplex biological networks by randomized optimization of modularity**,” Didier et al. apply a network clustering method to identify communities that are significantly enriched in disease signatures. They build on their previously published community detection method, which is based on the greedy optimization (following the algorithm by Louvain et al.) of multiplex modularity. The study is submitted as part of the DMI DREAM Challenge, which aims to evaluate different clustering algorithms by how well the resulting clusters are associated with GWAS-implicated disease genes. The authors test their approach on simulated datasets as well as DMI benchmark datasets. They have three improvements on their original method: randomization, edge and layer weights, and recursive clustering for large clusters.

 The DMI DREAM Challenge itself is an important exercise addressing the non-trivial task of identifying biologically meaningful communities in molecular networks. This paper by Didier et al. takes on this challenge by treating the benchmark datasets as a multiplex network. While limited by the constraints of the challenge, I think the paper is an important contribution that offers an insight as to how multiplex methods fare on real-world biological networks of diverse and not necessarily related sources.

 The paper is well written, but it has room for improvement, especially in the concise way information is presented in the Methods and Results section. Most of my major comments relate to the clarification of details I thought to be missing from Methods and Results. In various places in the manuscript, the reader is assumed to be familiar with the DREAM Challenge and the previous paper [5] on which this paper is based. The Methods section should thus be expanded to render the study more accessible and self-contained. In some places, the results could be interpreted better. Below are my suggestions meant to help the authors improve the presentation of their study:

**Major comments:**

 1) Randomly generated synthetic networks: Even if previously published in [5], it would be helpful if this were described a little more in detail. Providing details about how the community structure is defined or what exactly balanced communities means would be helpful to the reader.

 2) More information is needed for the network types, even if they’re described elsewhere under the umbrella of the DMI challenge. What were the sources for each network? Which organism are the PPI networks derived from? Are the co-expression networks tissue-specific or not?  Details like these would be informative when interpreting the results of Figure 3 from a biological standpoint.

 3) It is important to clarify how the multiplex network was constructed out of the six networks. For it to be a multiplex network, the same set of nodes should be represented on each layer, which likely requires the pruning of the six benchmark networks. How many nodes did the final multiplex consist of? How many edges were on each layer? Is there any specific inter-layer link structure?

 4) The authors use PASCAL to determine the disease-associated genes from GWAS, which are in turn used to determine the communities that are significantly enriched in disease signals. It is crucial at this point to convey to the reader very clearly what PASCAL does and how it’s translated to “significant disease modules.” The authors should, with a couple of sentences, describe how PASCAL does the SNP-to-gene mapping, and then, more importantly, how the p-value weighted gene annotations from PASCAL are used in the enrichment to determine the significant disease modules. A good description of this would facilitate the interpretations of Figure 2-4.

 5) The Methods section on controlling the size of modules could be more informative. “We tested different pre-filters (pruning leaves), parameters (resolution parameter, recursions, combination of graph weights for multiplexes) and post-filters (density, size, pruning leaves) in each leaderboard round.” This sentence contains a lot of information without really getting into details. The authors could elaborate on each item. For instance, the resolution parameter does not really seem to come into play (it is even omitted from the definition of multiplex modularity since it is set to 1). Recursion was used to limit the size of the clusters, and while the authors mention tests conducted in leaderboard rounds, no data here is shown as to how varying the resolution parameter changes the results. Perhaps the authors could either omit the section about the resolution parameter altogether or provide some supplementary figures on it.

 6) The 100 cutoff is set by the challenge, but in other settings, is there a way to set this ad hoc limit more concretely? Could the authors comment on this?

 7) In Figure 1, the authors note that the randomizations improve the accuracy of the detected communities, in particular for dense multiplex networks. This is interesting. May that suggest that dense networks have more possibilities whereby local maxima in the modularity landscape can lead to better results than the best solution? Can the authors comment on possible reasons why this could be the case?

 8) What Figure 2 tells me is that if we simply relax the association criterion (FDR cutoff), we’ll simply get more enriched communities, which is not entirely surprising. Here I think the distinction between the different GWAS datasets is important to discuss, if the authors are saying the results are sensitive to the datasets. What is it that makes the “Leaderboard” and “Final” datasets different? What are the diseases and traits included in the GWAS datasets? Perhaps the authors could comment on this.

 9) Also for Figure 2 (I may be missing something obvious here) – are these results based on multiplex or monoplex?

 10) “We hypothesize that the community structures of these networks (if they exist) are so unrelated that it is pointless to seek for a common structure by integrating them.” This is an interesting point that should remind us that the multiplex biological network should be constructed with a hypothesis in mind (e.g. various molecular levels from transcripts to proteins to metabolites) rather than piling up different networks into a multiplex structure. There is not much reason to think that just adding different sources of biological information should increase the performance of detecting disease modules. The tradeoff between signal and noise with the addition of diverse biological layers can be added as a point of discussion.

 11) Multiplex versus monoplex: The notion that multiplex networks help identify a greater number of significant disease modules than monoplex networks combined is an exciting prospect. However, the evidence shown in Figure 3 is a little scant. How was the comparison done exactly? Were the number of significant modules from each separate network summed up and compared to the one from multiplex? These kinds of details should be included.

 12) I found the Discussion a bit too DREAM Challenge-oriented. I think the parts comparing the results with those of the top performer are unnecessary (but this may be a requirement of the challenge so ignore if that's the case). The authors should revise the discussion to recapitulate and interpret their results, and to highlight the advantages of using the multilayer approach. I think it is commendable that the authors chose to include all of the six datasets, regardless of how related they are. Even the fact that the addition of some (possibly orthogonal) layers is detrimental to the outcome is an important finding.

   

**Minor points:**

 1) In the definition of multiplex modularity: K^(g)_i should be lowercase, i.e. k^(g)_i.

 2) Also in the definition of multiplex modularity, I would suggest the authors use s_i instead of k_i if they’re dealing with weights to denote the strength of the node rather than the degree. This is an optional point, but it would make it more aligned with the network science literature and nomenclature.

**Typos:**

 - “biological networks are nowadays assembled on a large-scale.” --> on a larger scale

 - “picks the move that increases the most modularity” --> picks the move that increases modularity the most

 - “data in which we randomly withdrawn vertices” --> data in which we randomly withdraw (or just say remove) vertices

 - “but to a limited extend after more than four runs” --> to a limited extent

 - “obtained by considering or not these weights in the MolTi partitioning” --> obtained by considering and not considering these weights in the MolTi partitioning

I have read this submission. I believe that I have an appropriate level of expertise to confirm that it is of an acceptable scientific standard, however I have significant reservations, as outlined above.

# Response

**Published:** 2018-10-26

**Authors:** Anais Baudot

**Affiliations:** CNRS, France

**In their manuscript entitled “Identifying communities from multiplex biological networks by randomized optimization of modularity,” Didier et al. apply a network clustering method to identify communities that are significantly enriched in disease signatures. They build on their previously published community detection method, which is based on the greedy optimization (following the algorithm by Louvain et al.) of multiplex modularity. The study is submitted as part of the DMI DREAM Challenge, which aims to evaluate different clustering algorithms by how well the resulting clusters are associated with GWAS-implicated disease genes. The authors test their approach on simulated datasets as well as DMI benchmark datasets. They have three improvements on their original method: randomization, edge and layer weights, and recursive clustering for large clusters.**

**The DMI DREAM Challenge itself is an important exercise addressing the non-trivial task of identifying biologically meaningful communities in molecular networks. This paper by Didier et al. takes on this challenge by treating the benchmark datasets as a multiplex network. While limited by the constraints of the challenge, I think the paper is an important contribution that offers an insight as to how multiplex methods fare on real-world biological networks of diverse and not necessarily related sources.**

**The paper is well written, but it has room for improvement, especially in the concise way information is presented in the Methods and Results section. Most of my major comments relate to the clarification of details I thought to be missing from Methods and Results. In various places in the manuscript, the reader is assumed to be familiar with the DREAM Challenge and the previous paper [5] on which this paper is based. The Methods section should thus be expanded to render the study more accessible and self-contained. In some places, the results could be interpreted better. Below are my suggestions meant to help the authors improve the presentation of their study:**

We agree that our manuscript requires familiarity with the DMI DREAM challenge data and consortium manuscript: It is indeed not independant, and was specifically written as a companion paper to be published with papers from other participants for the F1000 dream challenge channel. We apologize for it’s strong size constraints, and the fact that we tried to avoid any overlap with the main consortium paper, available in bioRxiv and considered for publication in a journal. We checked and tried to give the same amount of details as the other papers published in DREAM channels, in particular in the DMI channel.

**Major comments:**

**1) Randomly generated synthetic networks: Even if previously published in [5], it would be helpful if this were described a little more in detail. Providing details about how the community structure is defined or what exactly balanced communities means would be helpful to the reader.**

This point was also stated by another reviewer, and we now have extended the method section describing the generation of random network with a known community structure. We simulated networks with 20 balanced communities, meaning that the communities have the same size (regarding the number of nodes).

**2) More information is needed for the network types, even if they’re described elsewhere under the umbrella of the DMI challenge. What were the sources for each network? Which organism are the PPI networks derived from? Are the co-expression networks tissue-specific or not? Details like these would be informative when interpreting the results of Figure 3 from a biological standpoint.**

We agree that more information about the individual networks can help deepening the understanding of some of the results presented in the article, but the networks are an important point and deeply described in the main DMI challenge consortium paper available on biorXiv. This challenge paper is still considered for publication in a journal, and we are expected to avoid any overlap. We however now state in the method section that all the data are human.

**3) It is important to clarify how the multiplex network was constructed out of the six networks. For it to be a multiplex network, the same set of nodes should be represented on each layer, which likely requires the pruning of the six benchmark networks. How many nodes did the final multiplex consist of? How many edges were on each layer? Is there any specific inter-layer link structure?**

This is a very important and interesting point. The integration of biological networks obtained from different sources into a multiplex network is indeed not straightforward, as the different networks contain different data. In the multiplex framework, it is not mandatory to prune the networks to get the same set of nodes: multiplex networks share the same set of vertices (i.e., the union of all the nodes from all layer), as defined in [1]. Each layer provides information for a subset of the nodes, and the information for the remaining nodes is unknown and considered as missing data. In this context, the number of nodes to be clustered is the union of nodes in each layer, not the intersection. It is also in this context that we tested network layers with missing data in the simulations.

The multiplex modularity is equal to the sum of the individual modularities of the graphs of the multiplex with regard to the same community partition. Therefore, there is no need for an inter-layer link structure when constructing the multiplex network.                                                         

                            

[1] Kivelä M, Arenas A, Barthelemy M, Gleeson JP, Moreno Y, Porter MA. 2014. Multilayer networks. Journal of Complex Networks 2:203–271

**4) The authors use PASCAL to determine the disease-associated genes from GWAS, which are in turn used to determine the communities that are significantly enriched in disease signals. It is crucial at this point to convey to the reader very clearly what PASCAL does and how it’s translated to “significant disease modules.” The authors should, with a couple of sentences, describe how PASCAL does the SNP-to-gene mapping, and then, more importantly, how the p-value weighted gene annotations from PASCAL are used in the enrichment to determine the significant disease modules. A good description of this would facilitate the interpretations of Figure 2-4.**

We rephrased the corresponding method section to mention p-value and multiple testing corrections. However, we have for this companion paper a strong size constraints. The PASCAL tool is a complex approach deeply described in a publication (Lamparter et al. Plos Computational Biology), and its application and use in the context of the DMI dream challenge is fully described in the consortium paper. A precise and correct description of the evaluation procedure, from SNP mapping to significant modules would be too long and overlapping with the consortium and PASCAL papers.

**5) The Methods section on controlling the size of modules could be more informative. “We tested different pre-filters (pruning leaves), parameters (resolution parameter, recursions, combination of graph weights for multiplexes) and post-filters (density, size, pruning leaves) in each leaderboard round.” This sentence contains a lot of information without really getting into details. The authors could elaborate on each item. For instance, the resolution parameter does not really seem to come into play (it is even omitted from the definition of multiplex modularity since it is set to 1). Recursion was used to limit the size of the clusters, and while the authors mention tests conducted in leaderboard rounds, no data here is shown as to how varying the resolution parameter changes the results. Perhaps the authors could either omit the section about the resolution parameter altogether or provide some supplementary figures on it.**

Indeed, the resolution parameter was missing in the equation, and we now have included it. In addition, we removed from the manuscript the sentence describing the various test we made as this was indeed possibly confusing.

Concerning the choice of the default resolution parameter, we made extensive tests that are provided below, but we lack space in the manuscript to detail them. These tests show that the recursion approach provides overall a higher number of significant disease modules in the Dream Module Identification (DMI) challenge framework.

Total number of modules obtained when running Molti with 10 randomizations on the 6 DMI challenge network for different values of the resolution parameter:

Table 1

Number of significant Modules obtained with variations of Gamma:

Table 2

Comparison with recursion (apply Molti in a recursive way to communities larger than 100):

Table 3

                                                    

For FDR= 0.1

Figure 1

For FDR = 0.05

Figure 2

For FDR = 0.01

Figure 3

**6) The 100 cutoff is set by the challenge, but in other settings, is there a way to set this ad hoc limit more concretely? Could the authors comment on this?**

Defining the optimal module size is a complex task. One could imagine using the challenge benchmark to find the optimal module size. However, this is not feasible in practice as 1) the computation of PASCAL scores are time-consuming and cannot really be used to optimize the algorithms, 2) the sensitivity/volatility of the results to the number of submitted modules and GWAS data used make the interpretation on a fine-grained scale difficult.

The consortium paper discuss this point and identify, in particular, that the average module size do not correlate with the number of significant modules identified by the different approaches (Figure 3C in bioRxiv). It is also interesting to note that some modules are submodules of larger modules.

**7) In Figure 1, the authors note that the randomizations improve the accuracy of the detected communities, in particular for dense multiplex networks. This is interesting. May that suggest that dense networks have more possibilities whereby local maxima in the modularity landscape can lead to better results than the best solution? Can the authors comment on possible reasons why this could be the case?**

This is a very interesting point. We were actually surprised by the extent to which randomization improves the community detection on simulated networks. We initially thought that the greater increase in the accuracy of the communities detected on dense multiplex networks came from the fact that there was more room for improvement in this case. Your point makes sense since one can expect a greater number of local maxima for the modularity for dense network than for sparse ones, in addition to more possibilities of moving vertices during the Louvain execution. This point definitely deserves to be further investigated.

**8) What Figure 2 tells me is that if we simply relax the association criterion (FDR cutoff), we’ll simply get more enriched communities, which is not entirely surprising. Here I think the distinction between the different GWAS datasets is important to discuss, if the authors are saying the results are sensitive to the datasets. What is it that makes the “Leaderboard” and “Final” datasets different? What are the diseases and traits included in the GWAS datasets? Perhaps the authors could comment on this.**

The number of significant communities increases when the FDR cutoff is larger, as expected. However, the results are also quite sensitive to the dataset considered as pointed out by the reviewer. The different GWAS datasets were chosen by the DREAM challenge organizers, and splited into “Leaderboard” and “Final” manually. A detailed discussion of the diseases and traits included in the GWAS datasets, as well as their comparison, is proposed in the DMI consortium manuscript (in particular in Figures 1, 3 and 4) and cannot be reproduced here.

**9) Also for Figure 2 (I may be missing something obvious here) – are these results based on multiplex or monoplex?**

In order to clarify this point, we now state in the figure caption that the results are based on the multiplex networks.

**10) “We hypothesize that the community structures of these networks (if they exist) are so unrelated that it is pointless to seek for a common structure by integrating them.” This is an interesting point that should remind us that the multiplex biological network should be constructed with a hypothesis in mind (e.g. various molecular levels from transcripts to proteins to metabolites) rather than piling up different networks into a multiplex structure. There is not much reason to think that just adding different sources of biological information should increase the performance of detecting disease modules. The tradeoff between signal and noise with the addition of diverse biological layers can be added as a point of discussion.**

Thanks for this interesting remark. We agree that multiplex biological networks should be constructed with a hypothesis in mind, and that the network that are piled up in a multiplex are expected to be realisations of the true underlying biological modules. This is the case for the two PPI network and the pathway network that are expected to represent cellular processes. In addition, pairs of proteins belonging to the same pathway or co-expressed are more likely to physically interact than pairs of random proteins [1]. Therefore, even if the biological information contained in co-expression or pathways networks is completely different to the one contained in PPI networks, they still can improve the detection of the communities by adding more signal to the system and reducing the bias and noise of individual networks as we previously demonstrated [2]. The cancer network, contrarily, might contain very different biological information.

However, concerning the bias and noise present in the different networks, as the bias and noise are not the same, we see the combination of the different sources in a more positive way, as not adding noise but complementing each other to reduce noise. For instance, if we take individual PPI networks obtained from different sources and we integrate them into a multiplex PPI network, we expect to obtain better clustering results than with the individual networks alone. All these monoplex PPI networks try to reflect the real PPI network containing all the human genes, all their interactions and their real community structure. However, they have missing data and they strongly depend on the technologies used to get the interactions (bias and noise). Therefore, since all of them attend to reflect the same system, the biological signal increases and the noise and missing data tend to reduce. We added a sentence to the discussion concerning this point.

[1] Rual JF, Venkatesan K, Hao T, et al.: Towards a proteome-scale map of the human protein-protein interaction network. Nature. 2005; 437(7062): 1173–1178.

[2] Didier G, Brun C, Baudot A: Identifying Communities from Multiplex Biological Networks. PeerJ. 2015; 3: e1525.

**11) Multiplex versus monoplex: The notion that multiplex networks help identify a greater number of significant disease modules than monoplex networks combined is an exciting prospect. However, the evidence shown in Figure 3 is a little scant. How was the comparison done exactly? Were the number of significant modules from each separate network summed up and compared to the one from multiplex? These kinds of details should be included.**

The comparison between the different approaches is done following the DMI challenge benchmark, that is by computing the number of significant disease modules (with a FDR threshold of 0.1). We computed this number of significant modules identified from the 6 biological networks independently, and then from combination of networks thanks to the multiplex framework . We used combinations of 2 to 6 networks, considered as multiplex networks of 2 to 6 layers.

**12) I found the Discussion a bit too DREAM Challenge-oriented. I think the parts comparing the results with those of the top performer are unnecessary (but this may be a requirement of the challenge so ignore if that's the case). The authors should revise the discussion to recapitulate and interpret their results, and to highlight the advantages of using the multilayer approach. I think it is commendable that the authors chose to include all of the six datasets, regardless of how related they are. Even the fact that the addition of some (possibly orthogonal) layers is detrimental to the outcome is an important finding.**

The manuscript is indeed DREAM challenge oriented as it is not an independent manuscript but a companion paper. Regarding the interpretation of the multiplex approach, we think it is important to state that the top-performers selected a subset of the 6 networks, reflecting also the reviewer’s comment on the choice of the network to be piled up. We agree that a lot could still be discussed regarding our (and others) obtained results in this challenge, and extended a bit the discussion. This point is also discussed in the challenge consortium manuscript.

**Minor points:**

**1) In the definition of multiplex modularity: K^(g)_i should be lowercase, i.e. k^(g)_i.**

Thanks for pointing this out, it has been changed to s following your next comment.

**2) Also in the definition of multiplex modularity, I would suggest the authors use s_i instead of k_i if they’re dealing with weights to denote the strength of the node rather than the degree. This is an optional point, but it would make it more aligned with the network science literature and nomenclature.**

Done

**Typos:**

Thanks for pointing all these typos !

**- “biological networks are nowadays assembled on a large-scale.” --> on a larger scale**

Done

**- “picks the move that increases the most modularity” --> picks the move that increases modularity the most**

Done

**- “data in which we randomly withdrawn vertices” --> data in which we randomly withdraw (or just say remove) vertices**

Done

**- “but to a limited extend after more than four runs” --> to a limited extent**

This sentence has been removed after other reviewer’s comment.

**- “obtained by considering or not these weights in the MolTi partitioning” --> obtained by considering and not considering these weights in the MolTi partitioning**

Done

# Peer review: Referee response for version 1

**DOI:** 10.5256/f1000research.16880.r36848

**Published:** 2018-08-17

**Authors:** Yasir Suhail

**Affiliations:** 1 Department of Biomedical Engineering, Yale University, New Haven, CT, USA

**Overview**

 The paper presents:

 1. a couple of improvements over the method in the authors' previous 2015 paper on multiplexed modularity, and

 2. the application of this improved method to the Disease Module Identification DREAM challenge.

**Method**

 The paper adequately describes the incremental improvement of the general method over the previous paper regarding the following.

 1. The algorithm randomly selects one of the moves improving the modularity at every stage. I assume this helps the method to arrive at different local minima for each run, thereby improving its performance on the nonlinear modularity surface.

 2. The incorporation of edge and graph weights into the definition of modularity.

 Both of these points are well justified, and presented in adequate detail. The only minor detail that is missing is that Equation 1 does not include the resolution parameter.

**Results and Performance Evaluation**

 The improvement in performance due to randomization is presented in Figure 1, while Figure 4 presents evidence of improvement due to the graph (layer) weights.

 The improvement due to edge weights is not presented, but it logically follows that any network model wherein edges with larger weights are likely to form within modules will show improved performance under a weighted definition of modularity.

 The authors did not have control over the DREAM challenge evaluation, but a reader might have a few questions regarding the performance evaluation. For example, if the judging criterion is the number of significantly GWAS associated modules, does the score improve by dividing one large disease associated module into two, if they both are still significant? 

**Presentaion**

 Other than the missing resolution parameter, the method is adequately presented. There were a few minor issues with language. For example:

 1. The second paragraph of the abstract should end with "the number of trait and disease communit ***ies*** detected."

 2. While describing the method of simulating the missing data, another form of the word should be used instead of " ... we randomly ***withdrawn*** vertices of each layer ..."

 The authors provide source code for both the latest version, and also the version that was used for the DREAM challenge. It would be helpful if the authors also provide, if those are readily available, the scripts that were used to run the DREAM and simulated data for the results presented. This will help answer minor questions regarding details like filtering etc.

**Conclusion and suggestions**

 I think the method described by the paper is scientifically valid and its presentation is adequate, other than the minor issues raised above.

 Some of the important questions that may be pursued further are related to the selection of the graph weights. Currently, the weights are selected arbitrarily using the relative performance of the individual networks on predicting the disease modules. Could there be a more systematic manner of weight selection based on both the congruency with what's known of the true modules and the relative redundancy in the various layers?

I have read this submission. I believe that I have an appropriate level of expertise to confirm that it is of an acceptable scientific standard.

# Response

**Published:** 2018-10-26

**Authors:** Anais Baudot

**Affiliations:** CNRS, France

**The paper presents:**

**1. a couple of improvements over the method in the authors' previous 2015 paper on multiplexed modularity, and**

**2. the application of this improved method to the Disease Module Identification DREAM challenge.**

****

**Method**

****

**The paper adequately describes the incremental improvement of the general method over the previous paper regarding the following.**

**1. The algorithm randomly selects one of the moves improving the modularity at every stage. I assume this helps the method to arrive at different local minima for each run, thereby improving its performance on the nonlinear modularity surface.**

**2. The incorporation of edge and graph weights into the definition of modularity.**

****

**Both of these points are well justified, and presented in adequate detail. The only minor detail that is missing is that Equation 1 does not include the resolution parameter.**

Thanks for pointing out this omission. The resolution parameter has been included to the Equation 1 in the revised version of the manuscript.

**Results and Performance Evaluation**

****

**The improvement in performance due to randomization is presented in Figure 1, while Figure 4 presents evidence of improvement due to the graph (layer) weights.**

****

**The improvement due to edge weights is not presented, but it logically follows that any network model wherein edges with larger weights are likely to form within modules will show improved performance under a weighted definition of modularity.**

The tests of edge weights is indeed not presented as a figure, but some data are summarized as a table (Table 2).

**The authors did not have control over the DREAM challenge evaluation, but a reader might have a few questions regarding the performance evaluation. For example, if the judging criterion is the number of significantly GWAS associated modules, does the score improve by dividing one large disease associated module into two, if they both are still significant?**

This is a tricky question. Briefly, the DREAM evaluation uses the PASCAL tool to compute a score for each modules according to their enrichments in GWAS-significant genes. The number of significant disease module in a given partition is then computed according to an enrichment threshold, and considering correction for multiple testing. In this context, the total number of submitted valid communities is important, as submitting more modules will induce a stronger correction for multiple testing. It is thereby not straightforward to evaluate if dividing a large disease module into two will lead to two submodules both still significant, as this will also increase the correction for multiple testing. In other words, submitting more modules can affect badly the results even if without multiple testing correction it would have increased the number of significant disease modules. The PASCAL tool also takes into account the GWAS statistics to compute the significance, it is thus overall very hard to use the obtained discrete results (i.e., the number of significant disease modules) to tune/optimize slight variations of the approach, as the variability in the results can come from many factors. Some of these points are discussed in the DMI challenge manuscript as some of the top-performers of the subchallenge 1 selected the modules based on their sizes without working on the improvement of the algorithms *per se*.

**Presentaion**

****

**Other than the missing resolution parameter, the method is adequately presented. There were a few minor issues with language. For example:**

**1. The second paragraph of the abstract should end with "the number of trait and disease communit *ies* detected."**

Corrected

**2. While describing the method of simulating the missing data, another form of the word should be used instead of " ... we randomly *withdrawn* vertices of each layer ..."**

We changed the instances of “withdraw” to “remove”

**The authors provide source code for both the latest version, and also the version that was used for the DREAM challenge. It would be helpful if the authors also provide, if those are readily available, the scripts that were used to run the DREAM and simulated data for the results presented. This will help answer minor questions regarding details like filtering etc.**

The scripts used to run the DREAM challenge approaches are all available from the challenge webpages as a community resource.

**Conclusion and suggestions**

****

**I think the method described by the paper is scientifically valid and its presentation is adequate, other than the minor issues raised above.**

****

**Some of the important questions that may be pursued further are related to the selection of the graph weights. Currently, the weights are selected arbitrarily using the relative performance of the individual networks on predicting the disease modules. Could there be a more systematic manner of weight selection based on both the congruency with what's known of the true modules and the relative redundancy in the various layers?**

This is a very interesting question. As noted by the reviewer, we selected the weights based on the relative performance on the individual networks. However, this could not be done in a more systematic way, as true modules are not known (and in fact the challenge goal was to defined a benchmark as a proxy for these true modules). Another approach could be to weight the different layers based on our a priori knowledge/trust of the different layers. However, we have difficulties understanding how it would be possible in practice to weight according to the relative redundancy of the various layers.

# Peer review: Referee response for version 1

**DOI:** 10.5256/f1000research.16880.r35949

**Published:** 2018-08-07

**Authors:** Lenore J. Cowen

**Affiliations:** 1 Department of Computer Science, Tufts University, Medford, MA, USA

The authors explain the method that underlies their submission to the 2016 DREAM Disease Module Identification challenge. The authors only discuss their results from subchallenge 2; they should either say this is what they are going to do up front or also mention their performance on subchallenge 1.

 The paper is not self-contained, it already assumes some familiarity with the setup of the challenge; since this is being published in a collection on F1000 related to the dream challenge, perhaps that is appropriate, however I would have preferred if the authors had spent more time describing the challenge:  i.e. what does it mean in more detail to identify communities from the six biological networks jointly, rather than independently?

 While the authors release their code, which is commendable, there are not sufficient details in the text to completely understand their methods without returning to the code. For example, they talk about a resolution parameter *γ but* this parameter is defined nowhere in their paper: is this a parameter of the GenLouvain method to which they refer?

 The authors present a randomized algorithm which they run from 1 to 10 times, and return the partition with highest modularity, but figure 1 uses only 1 to 5 randomization runs. Furthermore, the authors do not explain how the parameter "5" or "10" is selected. There are other method details that involve parameters that are missing, for example in their set of simulated networks, it is impossible to discover how they set their parameters from just this writeup: They write "we simulated random multiplex networks with a fixed known community structure,... and various topological properties (i.e. dense/sparse/mixed, with/without missing data)" Is this the same collection of simulated networks that they generated in a previous paper (reference 5 that they cite?) if so, please say this explicitly. Even the writeup in reference 5 is somewhat sketchy, but it's better than what is here.

 The authors conclude that the power of including multiplex networks is dependent perhaps on the networks being generated from partial views of the same underlying community structure; when networks 5 and 6, which were very different were included, the performance of their method degraded, and this was found across the challenge in general.

I have read this submission. I believe that I have an appropriate level of expertise to confirm that it is of an acceptable scientific standard, however I have significant reservations, as outlined above.

# Response

**Published:** 2018-10-26

**Authors:** Anais Baudot

**Affiliations:** CNRS, France

**The authors explain the method that underlies their submission to the 2016 DREAM Disease Module Identification challenge. The authors only discuss their results from subchallenge 2; they should either say this is what they are going to do up front or also mention their performance on subchallenge 1.**

 We indeed focused on subchallenge 2. During the challenge leaderboard phase, our consortium tested many very different approaches on subchallenge 1, and we did not evaluate Molti thoroughly in this subchallenge. In addition, Molti was specifically designed to leverage multiplex networks. We added a sentence (highlighted in blue) to the manuscript to state this point more clearly.

**The paper is not self-contained, it already assumes some familiarity with the setup of the challenge; since this is being published in a collection on F1000 related to the dream challenge, perhaps that is appropriate, however I would have preferred if the authors had spent more time describing the challenge: i.e. what does it mean in more detail to identify communities from the six biological networks jointly, rather than independently?**

 We agree that our manuscript was specifically written for the F1000 dream challenge channel: it has strong size constraints and requires some familiarity with the challenge. The consortium manuscript is available in bioRxiv, and is still under consideration for publication in a journal. We wanted to avoid any potential overlap in the interpretation of the results in between the consortium paper and our manuscript. We finally also checked other papers published in DREAM channels and tried to give the same level of details. We however extended the description of the DREAM sub-challenge 2 in the main text.

**While the authors release their code, which is commendable, there are not sufficient details in the text to completely understand their methods without returning to the code. For example, they talk about a resolution parameter *γ but* this parameter is defined nowhere in their paper: is this a parameter of the GenLouvain method to which they refer?**

 Thanks for pointing this out. Indeed, we had forgotten the γ parameter and its description in the method section. The γ parameter is a resolution parameter allowing to tune the size of the obtained communities. Increasing this parameter allows reducing the size of the obtained communities. We now present the results obtained by tuning this parameter instead of applying the recursion in the answer to another referee. We also extended the description of the code in the github repo and the description of the simulated networks with SBM to answer the comment below.

**The authors present a randomized algorithm which they run from 1 to 10 times, and return the partition with highest modularity, but figure 1 uses only 1 to 5 randomization runs. Furthermore, the authors do not explain how the parameter "5" or "10" is selected. There are other method details that involve parameters that are missing, for example in their set of simulated networks, it is impossible to discover how they set their parameters from just this writeup: They write "we simulated random multiplex networks with a fixed known community structure,... and various topological properties (i.e. dense/sparse/mixed, with/without missing data)" Is this the same collection of simulated networks that they generated in a previous paper (reference 5 that they cite?) if so, please say this explicitly. Even the writeup in reference 5 is somewhat sketchy, but it's better than what is here.**

1. Number of randomizations

We agree that the different number of randomizations used along the manuscript can be confusing. We tried to make this point clearer in the revised version. The number of randomizations needed for a given dataset is not easy to set *a priori*. Since increasing the number of randomizations improves the modularity - and hopefully the accuracy - of the communities detected, we basically increased the number of randomization until obtaining no significant change in the communities detected. For the simulations, we selected 5 randomizations because the adjusted Rand index plot for 5 randomizations is confounded with that for 4 randomizations. For the Dream Challenge dataset, we considered 10 randomizations because increasing this number was too heavy in computation time.

1. Simulated networks with a community structure

The method section has been extended to describe SBM network simulations more precisely. These random networks are generated with the same protocol as the one described in reference 5, but were re-generated for this project. In addition, the code to generate the multiplex networks with SBM (and detect and compare the communities with adjusted Rand index) is now available on the github repository.

# Peer review: Referee response for version 1

**DOI:** 10.5256/f1000research.16880.r36392

**Published:** 2018-08-03

**Authors:** Emre Guney

**Affiliations:** 1 Research Programme on Biomedical Informatics, the Hospital del Mar Medical Research Institute, Pompeu Fabra University, Barcelona, Spain

Didier and colleagues present MolTi-DREAM, an update to their previous software for community detection in multiplex networks and its application to the disease module discovery using synthetic and DREAM challenge data. The new version of the software adds the possibility of edge and layer weights, the randomization of the underlying Louvain algorithm and partitioning of large modules into smaller modules based on user defined parameters. Based on their analysis of simulated and biological interaction data, they reaffirm that using multiple layers of networks and randomized repetition of module discovery could improve the accuracy. Overall the article is clearly written and the technical details are clearly explained with several exceptions I mention below:

 1. The multiplex modularity formula lacks the resolution parameter (which should appear before ki kj / 2m in the summation). The authors are encouraged to provide data / figures with respect to the reasoning on the selection of the default resolution parameter (currently they mention verbally that it showed better results on leaderboard).

 2. Randomization procedure is unclear. I believe the cost function used is the multiplex modularity given in Eq 1 (L) but the “move” leading to an increase of the modularity is not formally stated (sth like i, j: random_pick(argmax ci, cj L)). The choice of 4 by default is also somewhat arbitrary given that the difference between 3,4,5 are all very small. The significance of the difference in Rand index across runs in Figure 1 could be used to decide the number after which the change is not significant.

 3. The simulation of the multiplex networks could be explained better. Potentially owing to the previous publication, there is no detail in regards to which model was used to generate these networks. A brief description of SBMs and the underlying assumptions of the model it relies on would help. Are these simulated networks are more ER-like? Could this be the reason why the simulated results do not reflect the results on the real networks? Also, it would certainly be useful to add the standard deviation across 2000 networks in Figure 1. I had difficulties following how exactly the mixed and noisy networks are generated (did the mixed ones still have 1000 vertices, 500 from sparse and 500 from dense, for the missing data if the vertices were drawn half of the time, did they have 500 vertices?).

 4. The coverage / precision of disease module discovery is missing. What is the number of all modules identified by the algorithm, how many disease modules are there (or could there be) in total? The latter question could be tricky to answer but I believe a lower and upper bound could be provided (e.g., number of diseases and a number based on the GWAS hits for that disease divided by the min module size).

 5. “combining biological networks in a multiplex generally increases the number of significant modules”. The Figure 3 does not exactly reflect this, it seems the number of significant modules for multiplex(ppi1, ppi2) < monoplex(ppi1) + monoplex(ppi2). Maybe the authors could show the numbers for the union of the modules identified using ppi1and ppi2 and put that into the figure as to what to expect when the modules are combined using these networks separately.

 6. The choice of the for each biological network seems rather arbitrary. Could these weights be optimized (i.e. using leaderboard data) and their effect be tested on final data?

 7. “the community structure of these networks (if they exist) are so unrelated that it is pointless to seek for a common structure by integrating them” I feel that this statement should be supported by further evidence. Could the authors provide some measures in regards to the modularity of each of these networks and the overlap of nodes / edges between them. This would also help to justify the argument on the “DMI biological networks are constructed from different biological sources that might correspond to unrelated community structures”.

 Minor:

 Explain what monoplex network means at its first occurrence for readers not familiar with terminology.

 “hande mulxtiplexes” multiplex networks

 “following the PASCAL tool” identified using

 “Considering a greater number of layers...” lacks the main clause / verb

 “after more than four runs” upto four runs / repetitions

 “varies in a non-trivial way” non-trivial?

I have read this submission. I believe that I have an appropriate level of expertise to confirm that it is of an acceptable scientific standard, however I have significant reservations, as outlined above.

# Response

**Published:** 2018-10-26

**Authors:** Anais Baudot

**Affiliations:** CNRS, France

**1. The multiplex modularity formula lacks the resolution parameter (which should appear before ki kj / 2m in the summation). The authors are encouraged to provide data / figures with respect to the reasoning on the selection of the default resolution parameter (currently they mention verbally that it showed better results on leaderboard).**

Indeed, the resolution parameter was lacking in the formula. We now have corrected this. Concerning the choice of the default resolution parameter, we made extensive tests that are provided below, but we lack space in the manuscript to detail them. These tests show that the recursion approach provides overall a higher number of significant disease modules in the Dream Module Identification (DMI) challenge framework.

Total number of modules obtained when running Molti with 10 randomizations on the 6 DMI challenge network for different values of the resolution parameter:

Table 1

Number of significant Modules obtained with variations of Gamma:

Table 2

Table 3

Table 4

Comparison with recursion (apply Molti in a recursive way to communities larger than 100):

Table 5

  

For FDR= 0.1

Figure 1

For FDR = 0.05

Figure 2

For FDR = 0.01

Figure 3

**2. Randomization procedure is unclear. I believe the cost function used is the multiplex modularity given in Eq 1 (L) but the “move” leading to an increase of the modularity is not formally stated (sth like i, j: random_pick(argmax ci, cj L)). The choice of 4 by default is also somewhat arbitrary given that the difference between 3,4,5 are all very small. The significance of the difference in Rand index across runs in Figure 1 could be used to decide the number after which the change is not significant.**

The cost function is indeed the multiplex-modularity given in Eq 1. At each step, all the moves of an element from a community to another that lead to an increase of the multiplex-modularity are considered, and one of them is randomly picked with a uniform probability. The final number of randomizations was arbitrarily chosen, but several were tested during the Challenge.

The choice of 4 randomizations by default was indeed arbitrary, and we removed this sentence in the manuscript to just state that this number is user-defined. The significance of the difference in Rand indexes can also be used to select a correct number of randomizations. However, since the adjusted Rand index is computed with regard to the real communities, it could be used only when the real communities are known, i.e. in our random network simulations. For the real biological networks provided by the challenge, the actual community structure is unknown.

Increasing the number of randomizations cannot harm (except with regard to the computation time) since the modularity of the detected communities increases with this number. A possibility could be to test the significance of the modularity increase between two successive numbers of randomizations. However, computing this significance is complex and time-consuming for large networks.

**3. The simulation of the multiplex networks could be explained better. Potentially owing to the previous publication, there is no detail in regards to which model was used to generate these networks. A brief description of SBMs and the underlying assumptions of the model it relies on would help. Are these simulated networks are more ER-like? Could this be the reason why the simulated results do not reflect the results on the real networks? Also, it would certainly be useful to add the standard deviation across 2000 networks in Figure 1. I had difficulties following how exactly the mixed and noisy networks are generated (did the mixed ones still have 1000 vertices, 500 from sparse and 500 from dense, for the missing data if the vertices were drawn half of the time, did they have 500 vertices?).**

Indeed, the simulations have been described in our previous publication, but the current version was lacking details. We extended the method section to describe more precisely how the simulated networks with a known community structure, and their variations are generated.

As detailed in the revision, a key property of the SBMs is that, given a community structure, each edge is drawn independently with a probability that depends only the community to which its nodes belong. These property is certainly not granted in biological networks but random graph models taking into account dependance between edges (e.g., exponential random graph models) are difficult to estimate and to simulate. We considered simple SBMs parameterized with a probability for edges between nodes in a same community (intra-community edges) and a probability for edge between nodes belonging to two different communities (inter-community edges). Networks obtained from SBMs are generally not homogeneous, thus quite different from ER ones. The software performing the simulation of multiplex networks from SBMs, called “simul” is now available in the GitHub repository.

We tried to display the standard deviation of the adjusted Rand index, but the result was quite confusing since the the error bars tend to overlap themselves (see below for the simulated sparse and dense networks).

Figure 4

Figure 5

**4. The coverage / precision of disease module discovery is missing. What is the number of all modules identified by the algorithm, how many disease modules are there (or could there be) in total? The latter question could be tricky to answer but I believe a lower and upper bound could be provided (e.g., number of diseases and a number based on the GWAS hits for that disease divided by the min module size).**

Indeed, the total number of discovered modules was not detailed in the manuscript. It was however used by the PASCAL tool to compute the number of significant disease modules, as the total number of submitted modules is used to correct for multiple testing. We added the total number of discovered valid modules (of size [7-100]) to the figure and table captions.

Number of total modules detected by Molti for the results presented on:

- **Figure 2. The numbers used in the Figure 2  have been added to the caption.**

Table 6

- **Figure 3: **

Table 7

 

- **Table 2: (nRandom = 10). These numbers have been added to the Table 2 caption**

No Weights <- 615

Weights <- 585

- **Figure 4: (nRandom = 10).These numbers have been added to the Figure caption.**

No Weights <- 585

Confidence Weights <- 555

Inverse Confidence Weights <- 648

The question of how many disease modules exist or could exist is indeed tricky to answer, and is partly discussed in the DMI challenge consortium paper. In this challenge manuscript, the full detail on the 180 GWAS disease/trait datasets, their classification, as well as the scoring method are detailed. Briefly, a module is considered significant if it is enriched in at least one GWAS-associated trait or disease according to the PASCAL approach. A sampling approach is also used to account for robustness in comparing the challengers’ results. The figures 1, 3 and 4 of the consortium paper are in particular and attempt to dig in the reviewer’s proposed direction.

https://www.biorxiv.org/content/early/2018/02/15/265553

**5. “combining biological networks in a multiplex generally increases the number of significant modules”. The Figure 3 does not exactly reflect this, it seems the number of significant modules for multiplex(ppi1, ppi2) < monoplex(ppi1) + monoplex(ppi2). Maybe the authors could show the numbers for the union of the modules identified using ppi1and ppi2 and put that into the figure as to what to expect when the modules are combined using these networks separately.**

The modules identified by the multiplex approach cannot be directly compared to the sum of the modules identified independently in the monoplex networks, as the communities might be the sames or very similar. In principle, the two PPI networks should detect similar communities, since they are supposed to contain biological information of the same nature. However, both networks are built from different sources, each one with different features or missing data. Therefore, their combination into a multiplex network should lead to a closer representation of the “real PPI network”, and is expected to result in the detection of a larger number of more accurate communities. 

In order to compare the communities detected by the two monoplex PPI networks individually with the communities identified when these networks are integrated into a 2-layer multiplex network, we computed the adjusted Rand index  [1] between the three corresponding partitions. The adjusted Rand index is the corrected-for-chance version of the Rand index, which is a measure of the similarity between two data clustering [2].  It is usually applied to compare partitions obtained by different clustering algorithms on the same network. Nevertheless, it can be applied to our problem by considering the common nodes between every pair of monoplex network (i.e., considering the partitions reduced to the intersection of the two sets of elements).

The results are displayed below in a table and a figure:

**PPI_1 PPI_2 PPI1_+_PPI2**

**PPI_1** **1.0000000** 0.1075211 0.2645176

**PPI_2** 0.1075211 **1.0000000** 0.2906837

**PPI1_+_PPI2** 0.2645176 0.2906837 **1.0000000**

Figure 6

These results show that the community structure obtained from the multiplex approach is closer to the ones found with both single PPIs. In other words, the communities obtained from single PPI networks are closer to the multiplex than to the communities obtained from the other single network. In addition, 2*RandIndex(PPI_1,PPI_2) < RandIndex(PPI_1, **PPI1_+_PPI2**) and 2*RandIndex(PPI_1,PPI_2) < RandIndex(PPI_2, **PPI1_+_PPI2**). Therefore, it seems that the multiplex approach can reflect better the “real” community structure of the “real PPI” than the addition of both single networks. Finally, the community partition of the multiplex is closer to the one of the PPI_2 than to the one of the PPI_1.

**6. The choice of the for each biological network seems rather arbitrary. Could these weights be optimized (i.e. using leaderboard data) and their effect be tested on final data?**

We indeed considered different optimizations of the weights. This point was not clearly stated in the manuscript, but the weights were chosen based on the number of significant modules obtained in the application of Molti on the isolated monoplex networks. We rephrased the corresponding sentence to clarify this.

However, we do not think *a posteriori *that the results of the leaderboard can be used to choose the best weights in the final data : the results obtained using the Leaderboard and the Final GWAS datasets (as well as using the union in the Total GWAS dataset) are not clearly correlated, making difficult to optimize the weights from the Leaderboard results. In addition, the evaluation of the significance of the results by a discrete number of significant disease modules is strongly sensitive to the number of submitted modules, because of the correction for multiple testing. This volatility makes the benchmark difficult to use to evaluate subtle changes in the methods. That’s why we implemented and tested our approach on the simulated networks. Unfortunately, the simulated networks cannot be used to evaluate weights.

**7. “the community structure of these networks (if they exist) are so unrelated that it is pointless to seek for a common structure by integrating them” I feel that this statement should be supported by further evidence. Could the authors provide some measures in regards to the modularity of each of these networks and the overlap of nodes / edges between them. This would also help to justify the argument on the “DMI biological networks are constructed from different biological sources that might correspond to unrelated community structures”.**

The modularity is a measure that needs to be computed on a community structure, hence we need first to partition the networks to compute the modularity. To evaluate the modularity of the considered biological networks, we applied Molti with default parameters on the individual networks (as in Figure 3) to obtain the network partitions. We then computed the adjusted Rand index [1] in between the 6 network partitions. Here also, the adjusted Rand index is applied to our problem by considering the common nodes between every pair of monoplex network (i.e., considering the partitions reduced to the intersection of the two sets of elements).

The Adjusted Rand Index computed for every pair of partitions of the monoplex networks provided by Molti (10 randomizations) are displayed in the following table and figure:

**PPI_1  PPI_2  PATH_3  COEX_4 CANCER_5  HOMO_6**

**PPI_1** **1.000000000** 0.107521064 0.082823431 0.012937084 0.004922555 0.051958946

**PPI_2** 0.107521064 **1.000000000** 0.091792998 0.010104981 0.004755694 0.023984747

**PATH_3** 0.082823431 0.091792998 **1.000000000** 0.007815447 0.001958814 0.024252282

**COEX_4** 0.012937084 0.010104981 0.007815447 **1.000000000** 0.002040522 0.006049318

**CANCER_5** 0.004922555 0.004755694 0.001958814 0.002040522 **1.000000000** 0.002211310

**HOMO_6** 0.051958946 0.023984747 0.024252282 0.006049318 0.002211310 **1.000000000**

Figure 7

The two PPI networks are the most similar in terms of communities structure, as expected. The community structure of the Pathways network is also quite similar to both PPIs, which makes sense since proteins in the same pathway are more likely to interact physically than those belonging to different pathways. In addition, the Homology network is more similar to those three networks than the Co-expression network. This might come from the fact that this homology network is built by expanding known pathways (Kegg in particular) with the addition of new member related by eukaryotic homology, as detailed in the challenge consortium manuscript. The partition the most different is the one obtained from the cancer network.

[1] Santos J, Embrechts M. 2009. On the use of the adjusted rand index as a metric for evaluating supervised classification. In: Alippi C, Polycarpou M, Panayiotou C, Ellinas G, eds. Artificial neural networks—ICANN 2009. Lecture notes in computer science, vol. 5769. Berlin Heidelberg: Springer, 175–184.

 [2] W. M. Rand (1971). "Objective criteria for the evaluation of clustering methods". Journal of the American Statistical Association. American Statistical Association. 66 (336): 846–850. doi:10.2307/2284239. JSTOR 2284239.

Minor:

Explain what monoplex network means at its first occurrence for readers not familiar with terminology.

OK, done in the method section

“hande mulxtiplexes” multiplex networks

OK, all occurrences of “multiplexes” have been changed to “multiplex networks”

“following the PASCAL tool” identified using

Changed “following” to “using”

“Considering a greater number of layers...” lacks the main clause / verb

OK, changed to “We observed that considering a greater …”

“after more than four runs” upto four runs / repetitions

OK, changed to “Increasing the number of randomization runs improves the results up to four runs.”

“varies in a non-trivial way” non-trivial?

OK, we changed to “is strongly dependent“. This expression was related to the fact that we do not observe a clear correlation between the results obtained on the leaderboard and final GWAS datasets.
